# Savings Banks

**Published:** 1874-11

**Authors:** 


### SAVINGS BANKS.

In spite of all the haste to be rich, it is the careful and the saving who accumulate rather than the bold and speculative. It is said that among merchants in our large cities, only two out of every hundred are successful in accumulating wealth. To periodically put money at interest, is certain to lead to a competency in the course of an ordinary lifetime.

Mr. A. M. Lesley, President of the Trades Savings Bank, near the Grand Opera House on Twenty-third street, gives us some encouraging facts for consideration during this depressed financial period. Since June last the deposits in this bank have increased about 60 per cent. This looks as if economy and forethought were taking the place of the extravagance and recklessness which have been so prevalent since the war. The panic has taught a lesson which the wise will heed and remember. The extravagance and the speculative tendencies which brought such wide-spread ruin, are being superseded by a reduction of expenses and the storing up of something for a day of need. The salutary lesson of the bee, who while securing his daily bread contrives to lay up something for the future, is being imitated.

It is wonderful how rapidly interest accumulates. It is said to have been definitely ascertained that had two cents been put out at 6 per cent. interest in the year 1, A. D., the total value would now equal that of 25,000 solid globes of pure gold, each as large as this earth; an amount which all the banks in the world could not begin to pay the interest on.

Nearly all over the country there are good, carefully managed savings banks in which our readers can place their spare funds. In this city there are many excellent ones, and all of them passed safely through the late panic. We think the "Trades" is certain one day to occupy a leading position. So far it has been able to pay 7 per cent. per annum for all money deposited. This is an exceptionally liberal rate. The money deposited is only loaned on the best securities, under the supervision of the prominent and wealthy citizens who comprise the excellent Board of Trustees. The bank is kept open until 8 o'clock each evening for the convenience of those who wish to draw or deposit funds.

Remittances may be made by distant depositors through Post-office order or draft mailed to the bank, and the amount will be duly credited and the book returned by mail.

